# Supplementary material for: F‐actin patches associated with glutamatergic synapses control positioning of dendritic lysosomes
Source: EMBO J. 2019 Jun 27;38(15):e101183. doi: 10.15252/embj.2018101183 (PMC6669925; doi:10.15252/embj.2018101183)
Supplement: Supplementary file 1 — Appendix [file EMBJ-38-e101183-s001.docx]

**Appendix**

**F-actin patches associated with glutamatergic synapses control positioning of dendritic lysosomes**

Bas van Bommel^1*^, Anja Konietzny^1*^, Oliver Kobler^2^, Julia Bär^1^, Marina Mikhaylova^1#^

**Table of Contents**

Materials and Methods 1

Appendix Figure S1 11

Appendix Figure S2 13

Appendix Figure S3 14

Appendix References 15

**Materials and Methods**

**Constructs**

LAMP1 (transcript-variant x1; *XM_017599994.1*) sequence was amplified from a rat brain cDNA library. For generation of the library, RNA was extracted with an RNeasy Plus mini kit (*Qiagen*) according to the manufacture’s protocol and transcribed into cDNA using SuperScript III Reverse Transcriptase (*Invitrogen* #18080-093) and LAMP1-specific primers. The obtained sequence was ligated to eGFP (source: pEGFP-N1) and mCherry (source: pGolt-*mCherry, Addgene* #*73297*) plasmids by PCR. Then LAMP1-GFP or LAMP1-mCherry were subcloned into a pAAV2 (Mikhaylova et al, 2018) with EcoRI and HINDIII.

For cloning of the PEX3-EmeraldGFP-KIF17 construct the peroxisome targeting sequence of PEX3 (Accession NM_003630; previously described by (Kapitein et al, 2010)) was amplified via PCR and inserted into an empty pAAV2 vector using AgeI and HindIII restriction sites. GFP (mEmerald variant) was amplified via PCR and inserted behind the PEX3 via HindIII and BamHI restriction sites. The P2A sequence including a 3’ KpnI restriction site was amplified via PCR and inserted behind the GFP in the BamHI-cut vector using the Cold Fusion Cloning Kit (*SBI*, MC010B-1). The constitutively active KIF17 construct (β-actin KIF17(1-547)-FRB) was used as a PCR template and is a kind gift from Casper Hoogenraad and was described previously (Kapitein et al, 2013). KIF17 sequence was inserted behind the P2A sequence via KpnI and NheI restriction sites. The GFP-nanobody including the GSG9-linker (GSG9-vhhGFP4) was described before (Caussinus et al, 2011; Katrukha et al, 2017). The whole sequence including a 3’ NheI restriction site was amplified via PCR and inserted into the NheI-digested vector using the Cold Fusion Cloning Kit (*SBI*, MC010B-1). All constructs were verified by sequencing.

**Constructs**

| **Backbone** | **Promoter** | **Insert** | **Source** |
| --- | --- | --- | --- |
| pAAV | synapsin | mRuby2 | Gift from T.G. Oertner |
| pAAV | synapsin | mRuby3 | Subcloned from *Addgene* #74252 in pAAV backbone |
| pAAV | synapsin | YFP | (Mikhaylova *et al*, 2018) |
| pAAV | synapsin | LAMP1_(rat.trans.var.x1)_-eGFP | this study |
| pAAV | synapsin | LAMP1_(rat.trans.var.x1)_-mCherry | this study |
| pCAGGS | pCAG | FusionRed-actin_(NCBI:NM_031144.3)_ | Gift from A. Kostyukova |
| pAAV | synapsin | Actin-chromobody-TagRFP | Subcloned from *ChromoTek* into pAAV backbone |
| pCAG | pCAG | PSD95.FingR-eGFP-CCR5TC | *Addgene* #46295 |
| pCI | Synapsin | mCerulean | Gift from T.G. Oertner |
| pCI | Synapsin | myosinV-DN-mCerulean _(bp 4242 – 5487; NCBI: XM_006510832.3)_ | (González-Gallego *et al*, 2019) |
| pAAV | Synapsin | myosinVI-DN-GFP _(bp 3177-3789; NCBI: NM_001039546.2)_ | (González-Gallego *et al*, 2019) |
| pAAV | Synapsin | PEX3-emEGFP-p2A-KIF17 | this study |
| peGFP-C1 | CMV | eGFP | *Clontech #6085-1* |
| pcDNA3.1 | CMV | empty vector | *Thermo Fisher Scientific* #V855-20 |

**Reagents**

| **Reagent** | **Source** | **Product Number** |
| --- | --- | --- |
| LysoTracker Green DND-26 | *Thermo Fisher* | L7526 |
| LysoTracker Red DND-99 | *Thermo Fisher* | L7528 |
| phalloidin–Atto647N | *Sigma-Aldrich* | 65906 |
| DMSO | *Carl Roth* | 4720.4 |

**Antibodies**

*Primary antibodies*

| **Antibody** | **Species** | **Source** | **Product number** | **Dilution for immunostaining** | **Dilution for western blot** |
| --- | --- | --- | --- | --- | --- |
| bassoon | mouse | *Stressgen, now Enzo* | ADI-VAMPS003 | 1:500 | n/a |
| synaptotagmin 1  (lumenal domain) | rabbit | *Synaptic Systems* | 105 103C3 | 1:100 | n/a |
| homer1 | mouse | *Synaptic Systems* | 160011 | 1:500 | n/a |
| homer1 | rabbit | *Synaptic Systems* | 160103 | 1:500 | n/a |
| gepyhrin | mouse | *Synaptic Systems* | 147011 | 1:500 | n/a |
| GluNR1 | rabbit | *millipore* | AB9864 | 1:300 | n/a |
| GluA1 | rabbit | *Calbiochem* | PC246 | 1:300 | n/a |
| MAP2 (figure 3/S3) | mouse | *Sigma* | M4403 | 1:500 | n/a |
| MAP2 (figure S1) | rabbit | *Abcam* | Ab32454 | 1:400 | n/a |
| cortactin | rabbit | *Santa Cruz* | Sc-11408 | 1:200 | n/a |
| α-tubulin | mouse | *Sigma* | T5168 | 1:250 | n/a |
| LAMP1 | rabbit | *Abcam* | ab24170 | 1:200 | n/a |
| LAMP1 | mouse | *DSHB* | 1D4B | 1:400 | 1:1.000 |
| myoVa | rabbit | *Sigma* | m4812 | 1:400 | 1:1.000 |
| myoVI | rabbit | *Santa Cruz* | sc-50461 | 1:400 | 1:1.000 |
| KIF5C | rabbit | *Thermo Fisher* | PA1-644 | n/a | 1:1.000 |
| dynein IC1/2 | mouse | *Santa Cruz* | sc-13524 | n/a | 1:1.000 |
| PMP70 | mouse | *Sigma* | SAB4200181 | 1:750 | 1:1.000 |
| PEX14 | rabbit | *Proteintech* | 10594-1-AP | 1:750 | 1:1.000 |

*Secondary antibodies*

| **Antibody** | **Conjugate** | **Company** | **Product number** | **Dilution for immunostaining** | **Dilution for western blot** |
| --- | --- | --- | --- | --- | --- |
| Anti-mouse | Alexa Fluor 488 | Life Technologies | A11029 | 1:500 | n/a |
| Anti-rabbit | Alexa Fluor 568 | Life Technologies | A11036 | 1:500 | n/a |
| Anti-mouse | Abberior Star 580 | Sigma | 52403 | 1:250 | n/a |
| Anti-rabbit | Abberior Star 580 | Sigma | **41367** | 1:250 | n/a |
| Anti-mouse | Alexa Fluor 647 | Life Technologies | A21236 | 1:500 | n/a |
| Anti-mouse | HRP | Dianova | 115-035-146 | n/a | 1:20.000 |
| Anti-rabbit | HRP | Dianova | 111-035-144 | n/a | 1:20.000 |

**Pharmacological treatments**

Latrunculin A (LatA), SMIFH2, CK-666, Brefeldin-A, MyoVin, and TIP were dissolved in DMSO according to manufacturer’s recommendations. DIV18 neurons were treated with Brefeldin-A, LatA , SMIFH2, CK-666, or DMSO and after indicated time points were fixed in 4 % Roti-Histofix/4 % sucrose in phosphate buffered saline (PBS), and preceded for immunostainings as described in a later section. LatA treatment was initially performed for 15 and 35 min (Figure S1) and thereafter all other experiments were performed within this time window.

For myosin inhibitor treatments, DIV14 neurons were transfected with LAMP1-eGFP and mRuby3. One day after, 5-8 cells per condition were imaged before and after treatment with either MyoVin or TIP.

*Pharmacological compounds*

| **Pharmacological compounds** | **Source** | **Product number** | **Concentration** | **Treatment duration** |
| --- | --- | --- | --- | --- |
| TTX | *Tocris* | 1078 | 1 µM | 30 min |
| Latrunculin A | *Tocris* | 3973 | 5 µM | 30 min |
| Brefeldin A | *Cell Signalling* | 9972S | 100 ng/ml | 10 h |
| SIMFH2 | *Sigma-Aldrich* | S4826 | 30 µM | 90 min |
| CK666 | *Sigma-Aldrich* | SML0006 | 50 µM | 2 h |
| MyoVin | *Merck* | 475984 | 30 µM | 30 min |
| TIP | *Sigma-Aldrich* | 19566 | 4 µM | 30 min |
|  |  |  |  |  |

**Preparation of organotypic hippocampal slices and single cell electroporation**

Organotypic hippocampal slices were prepared from P5 rat pups (Wistar, *Envigo*). Hippocampi were dissected in ice-cold dissection solution (in mM: 248 sucrose, 26 NaHCO_3_, 10 D-glucose, 4 KCl, 5 MgCl_2_, 1 CaCl_2_, 2 kynurenic acid, 0.001 % phenol red – oxygenated with 95 % O_2_/ 5 % CO_2_) and sliced to 400 µm slices using a tissue chopper (*McIlwain*). Slices were cultured at 37 °C, 5 % CO_2_ on 0.4 µm millicell membranes (*Millipore*). Culture medium (80% HEPES-free MEM, 20 % heat inactivated horse serum, 200 mM HEPES, 1 mM L-glutamine, 0.00125 % ascorbic acid, 0.01 mg/ml insulin, 1.44 mM CaCl_2_, 2 mM MgCl_2_, 13 mM D-glucose – pH 7.38) was exchanged three times per week. For further information we refer to (Gee et al, 2017).

Single-cell electroporation was performed at DIV15 in sterile-filtered HEPES based extracellular solution (in mM: 145 NaCl, 10 HEPES, 25 D-glucose, 2.5 KCl, 1 MgCl_2_, 2 CaCl_2_ – pH 7.4). Plasmids for mRuby2 and PSD95.FingR_eGFP_CCR5TC were diluted in potassium based intracellular solution (in mM: 135 K-gluconate, 0.2 EGTA, 4 MgCl_2_, 4 Na_2_-ATP, 0.4 Na-GTP, 10 Na_2_-phosphocreatine, 3 ascorbate, 0.02 Alexa Fluor 594, 10 HEPES – pH 7.2) to a concentration of 40 ng/µl. Single cells were electroporated by applying 50 hyperpolarizing pulses (-12 mV, 0.5 ms, at 50 Hz) with an Axoporator 800A (*Molecular devices*). For an elaborate protocol we refer to (Wiegert et al, 2017). Slices were fixed with 4 % Roti-Histofix (*Carl-Roth*, A146.5)/ 4 % sucrose 3 days after electroporation, mounted in mowiol (as described in section Immunocytochemistry) and imaged with a confocal microscope.

**Synaptotagmin antibody uptake assay**

α-synaptotagmin-Oyster550 was diluted according to manufacturer’s protocol including glycerol to a concentration of 0.5 mg/µl. Primary neurons (DIV17) were incubated for 30 min with α-synaptotagmin-Oyster550 (1:100 in culturing medium). For synaptic silencing, 1 µM TTX was added directly before incubation with α-synaptotagmin-Oyster550. After incubation, neurons were washed in culturing medium (containing 1 µM TTX for silenced cultures) and fixed in 4 % Roti-Histofix / 4 % sucrose. The procedure was followed by immunostaining for homer and actin (phalloidin-Atto647N).

For quantification of synaptotagmin antibody uptake, raw confocal images were analysed using Fiji. The maxima function was used to identify homer spots. Manually, ROIs outside the main dendrite were removed. Circular ROIs with 15px (≈0.57 µm) radius were created and synaptotagmin integrated density measured within these ROIs.

**Lysosomal enrichment and characterization**

Enrichment of lysosomes was done using a Lysosome Enrichment Kit (*Thermo Fisher Scientific*, #89839) according to the manufacturer’s instructions. In brief, approximately 100 mg of tissue from the cortex and hippocampus of an adult, female rat was homogenized in buffer provided by the kit, using a Dounce homogenizer and cleared at 500 x g for 10 min at 4 °C. The supernatant was supplemented with 15 % OptiPrep and loaded on top of an OptiPrep gradient (five layers with 30 %, 27 %, 23 %, 20 % and 17 % OptiPrep) in an ultracentrifuge tube. Centrifugation was carried out at 145.000 x g in a swinging bucket rotor for 2 h at 4 °C. After centrifugation, the lysosome enriched band (upper-most band below the input) was removed and mixed with 3 volumes of PBS to decrease the concentration of the OptiPrep. The sample was centrifuged again at 18.000 x g for 30 min at 4 °C, the supernatant was removed and the lysosome pellet was either taken up in in sample buffer for SDS-PAGE and western blot, or resuspended in PBS, treated with 1:20.000 LysoTracker Red and then fixed for 1 h on ice with 4 % Roti-Histofix/4 % sucrose for immunostaining. For western blot analysis, lysosome samples were subjected to SDS-PAGE on a 4-20 % gradient gel and transferred to a PVDF membrane. The membrane was blocked (5 % milk in TBS + 0.1 % Tween-20) and stained with primary antibodies against myosin VI, myosin Va, dynein IC 1/2, and KIF5C, followed by HRP-coupled anti-mouse and anti-rabbit antibodies. The membranes were imaged on a ChemoCam imager (*Intas*). For immunostaining, coverslips were coated with poly-L-lysine (*Sigma-Aldrich*) for 4 h at RT and washed 5 times with milliQ H_2_O. 10 µl droplets with fixed lysosomes were put on top of a parafilm surface, then coated coverslips were placed on the droplets face-down and incubated for 25 min at RT. Afterwards coverslips were flipped over and washed 2 times with PBS. The surface was then blocked for 45 min with blocking buffer, washed 3 x with PBS, and stained with primary antibody (in blocking buffer; mouse anti-lamp1, rabbit anti-myosin VI, rabbit anti-myosin Va) for 1 h at RT (or overnight at 4 °C), washed 3 x in PBS, and stained with secondary antibody diluted in blocking buffer for 45 min at RT, washed 3 x in PBS and mounted in mowiol on a microscopy slide.

**Enrichment of microsome fraction from whole rat brain lysate**

The whole brain of a female adult rat was homogenized in 10 ml homogenization buffer (0.32 M sucrose, 5 mM HEPES, pH7.4, Roche complete protease inhibitors) per 1 g of tissue, using a Dounce homogenizer at 900 rpm (12 strokes). Homogenate was centrifuged for 10 min at 1000 g at 4°C. Supernatant (S1) was removed and kept on ice, the pellet (P1) was resuspended in 10 ml / g homogenization buffer and homogenized again for 12 strokes at 900 rpm. The homogenate was centrifuged at 100 g for 10 min at 4°C. Supernatant (S1’) was removed and combined with S1, pellet (P1’) was discarded. The combined supernatants (S1+S1’) were centrifuged at 12.000 g for 15 min at 4°C. Supernatant (S2) was removed and kept on ice, pellet (P2) was resuspended in 10 ml / g homogenization buffer and homogenized for 6 strokes at 900 rpm. The homogenate was centrifuged again at 12.000 g for 20 min at 4°C. Supernatant (S2’) was removed and combined with S2, pellet (P2’) was discarded. The combined supernatants (S2+S2’) were centrifuged at 100.000 g for 1 h at 4°C. The resulting pellet (enriched in microsomes) was resuspended in 1 ml homogenization buffer and kept on ice. For western blot analysis, 200 µl of the microsome fraction were mixed with 200 µl 2 x SDS-sample buffer, separated on a 4 – 20 % SDS-polyacrylamide-gel and blotted on a PVC membrane. The membrane was stained with primary antibodies against myosin Va(rb), PMP70(ms), PEX14(rb), and β-actin(ms), followed by HRP-coupled anti-mouse and anti-rabbit antibodies. The membranes were imaged on a ChemoCam imager (*Intas*). For immuno-staining, 200 µl of the microsome fraction were fixed by adding 400 µl fixation buffer (4% Roti-Histofix, 4 % sucrose in PBS) and kept over night at 4°C. For immunostaining, coverslips were coated with poly-L-lysine (*Sigma-Aldrich*) for 4 h at RT and washed 5 times with milliQ H_2_O. 10 µl droplets with fixed microsomes were put on top of a parafilm surface, then coated coverslips were placed on the droplets face-down and incubated for 45 min at RT. The coverslips were then washed for 3 x 5 min in PBS, blocked for 30 min in blocking buffer (10 % horse serum, 0.1 % Triton X-100 in PBS), and incubated over night with primary antibodes (Myosin Va(rb), Myosin VI(rb), PMP70(ms), PEX14(rb); 1:750 in blocking buffer) at 4°C. The coverslips were washed again for 3 x 10 min with PBS, stained for 1 h at RT with secondary antibodies (ms-alexa488, rb-alexa 568; 1:500 in blocking buffer), washed for 3 x 10 min with PBS and mounted on microscopy slides in mowiol.

**3D reconstructions in Imaris**

3D reconstructions were performed in Imaris v8.4.1 (*Bitplane*). A Gaussian blur filter of 1 px size was applied to the cell fill (mRuby2) channel of confocal z-stacks and afterwards dendrites were built using the *auto-path* option of the *filament tracer* with default settings. Puncta of FingR-PSD95-GFP and bassoon were detected using the *spots* function with manual thresholding in the corresponding channels.

**Analysis of average velocities inside and outside of actin patches**

The effects of actin patches on transport of LAMP1-positive vesicle and peroxisomes coupled to constitutively active KIF17 was analysed with a custom written Matlab script (*MathWorks*). Kymographs images for the F-actin channel were imported and a normalized intensity profile was generated from the first 100 time points. To reduce noise, the profile was filtered with a moving average filter. Actin patches were detected with the function *peak prominence*, the prominence was adjusted by the user and was dependent on the signal-to-noise level. Borders of the actin patches were defined as 60-75 % distance to the first minimum, separately for both slopes of the peak. To reduce experimental influences, a random control was generated. Borders of the actin patches were moved 5-15 µm (randomized) to the right. Patches that moved out of the kymograph area were added on the left site, so that the area on the kymograph covered by actin patches remained equal. Next, coordinates of displacements, manually traced in *Fiji*, were imported into Matlab. Average velocity of vesicles (including stationary) was computed for inside/outside actin patches for both the real data and randomized control. The velocity inside actin patches might be slightly overestimated, since the positions of actin patches are stated from the kymograph analyses as x, dendrite thickness, t. Vesicles passing by patches not directly interaction but elsewhere in the 3D volume are considered as inside patch regions. The analysis workflow is illustrated in Figure S2.

**Calculation of the directional net flux**

The directional net flux F is a measure for the summed transport direction of vesicles.

F = [Σ(r_anterograde_)- Σ (r_retrograde_)] / [Σ (r_anterograde_) + Σ (r_retrograde_)]

with r being run length

Outcomes range between -1 (exclusive retrograde movement) and 1 (exclusive anterograde movement), being 0 when anterograde matches retrograde movement.

**Cumulative pausing time**

As a measure for overall pausing behavior, the cumulative pausing time was calculated. All pausing times of all vesicles within one analyzed dendritic segment were summed over the complete imaging period before and after treatment (identical time) and normalized to the control group (before treatment) to obtain an n of 1.

**Lysosome count**

To compare the number of mobile vs. stationary LAMP1-positive organelles (lysosomes) in different treatment conditions, the same time-lapse movies and ROIs selected for kymograph analysis were used. All visible lysosomes in only the first frame of the time-lapse movie were counted manually (=total lysosomes). The number of stationary lysosomes is defined as those that stayed immobile during the whole imaging period (3 min), as visible in the kymograph. Mobile lysosomes are calculated as total lysosomes-stationary lysosomes.

**Analysis of actin patches**

The density of actin patches, size and mean fluorescence intensity of F-actin within given patch, presence of cortactin and association with homer1 were analyzed using Fiji. 2D STED images of dendritic stretches, 13 - 32 µm in length, were used for analysis. ROIs were drawn manually in the phalloidin channel (unprocessed STED) and subsequently; size, integrated and mean intensity and cortactin integrated density were measured in the unprocessed STED channels. Differentiation between spine and shaft associated was based on the proximity to clearly detectable dendritic spines (not further than 0.5 µm from the spine base). PSD positive or negative patches were defined by direct association with homer1.

**Appendix Figures:**


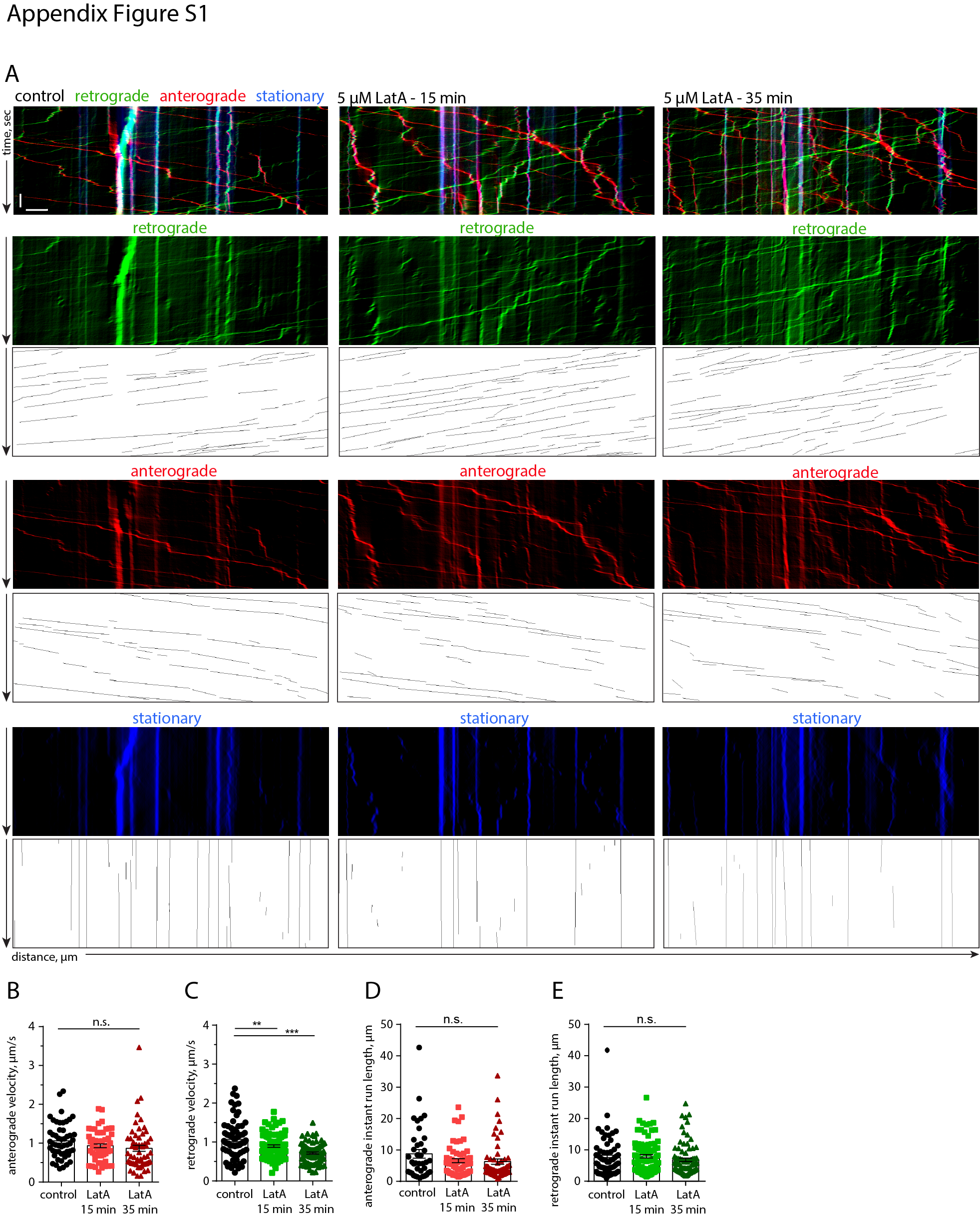


**Appendix Figure S1 - Workflow of kymograph analysis and quantification of additional lysosomal trafficking parameters.**

**A.** Representative kymographs from a DIV18 hippocampal neuron with LysoTracker Green before (control) and after 15 min or 35 min of 5 µM LatA treatment. Anterograde, retrograde, and stationary kymographs generated using the KymoGraphClear plugin for Fiji were traced manually. Scale bar: 5 µm.

**B-E.** Exemplary analysis of the kymographs traced in **A**. LatA treatment does not affect anterograde instant velocity (**B**). 1-Way-ANOVA. n=51 (control), n=57 events (LatA 15min, 35min). LatA treatment leads to a decreased retrograde velocity already after 15 min and 35 min (**C**). 1-Way-ANOVA. p<0.0001 with Dunnett’s post hoc test. ** p=0.005, *** p<0.0001. *n*=65 (control), *n*=90 (latA15min), *n*=81 events (LatA 35min). LatA treatment does not affect anterograde instant run length (**D**). 1-Way-ANOVA. Same n as in **B**. LatA treatment does not affect instant retrograde run length (**E**). 1-Way-ANOVA. Same *n* as in **C**. Data are presented as mean ± SEM.


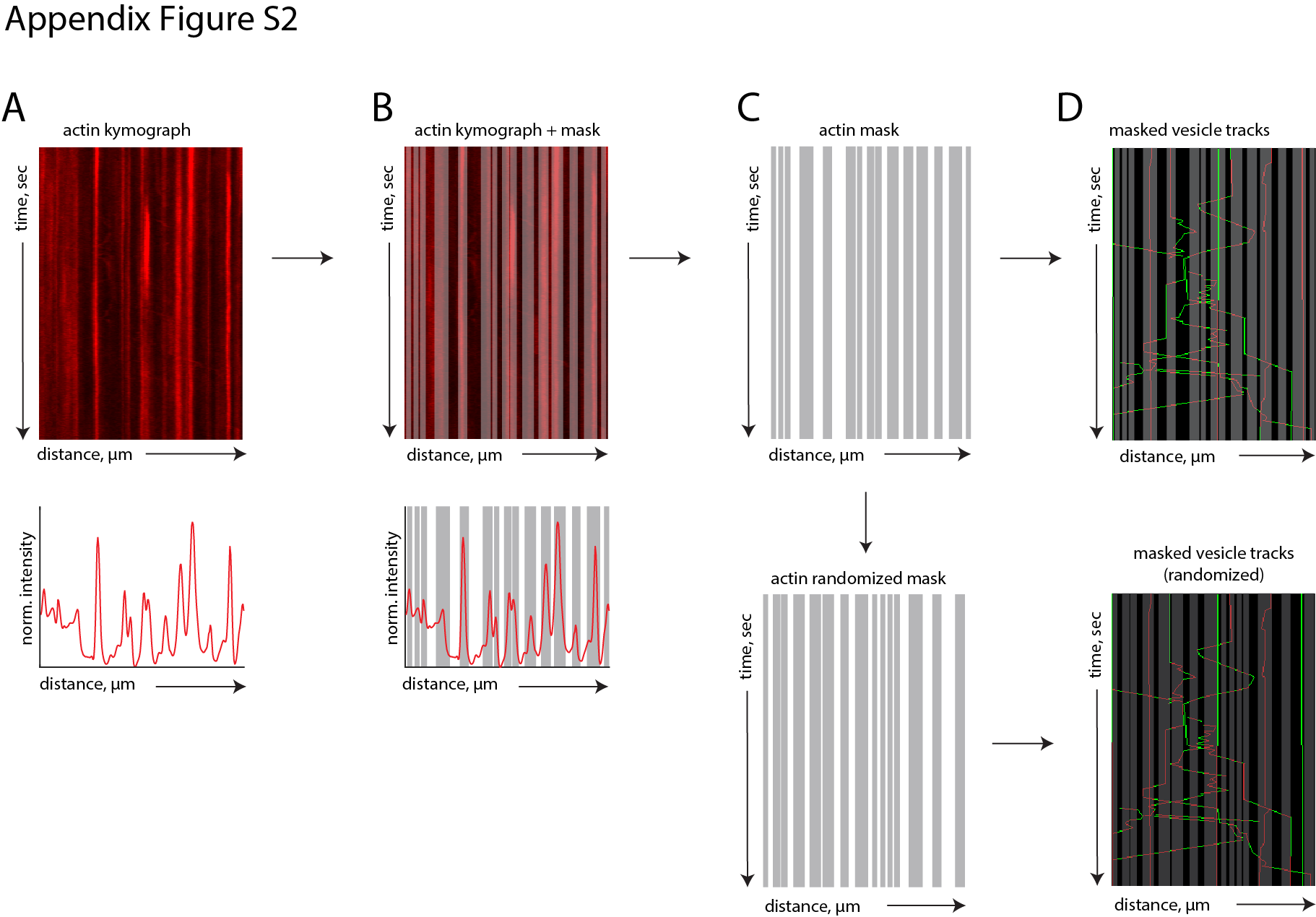


**Appendix Figure S2 – Workflow of the Matlab script developed for the analysis of organelle trafficking in specified areas of a dendrite.**

**A**. Kymograph of the F-actin channel and the corresponding normalized intensity profile plot of the first 100 time points.

**B**. Generation of a mask based on the plot profile of the actin patches.

**C**. The extracted actin mask. Additionally, the mask is randomized to create a control. The randomized control covers a same sized area.

**D**. Kymograph traces (traced by hand) are imported as lines. The overall average speed for vesicles is computed inside (red) and outside (green) of actin patches based on the actin mask (**C**) and the mask for the randomized control (**C**).


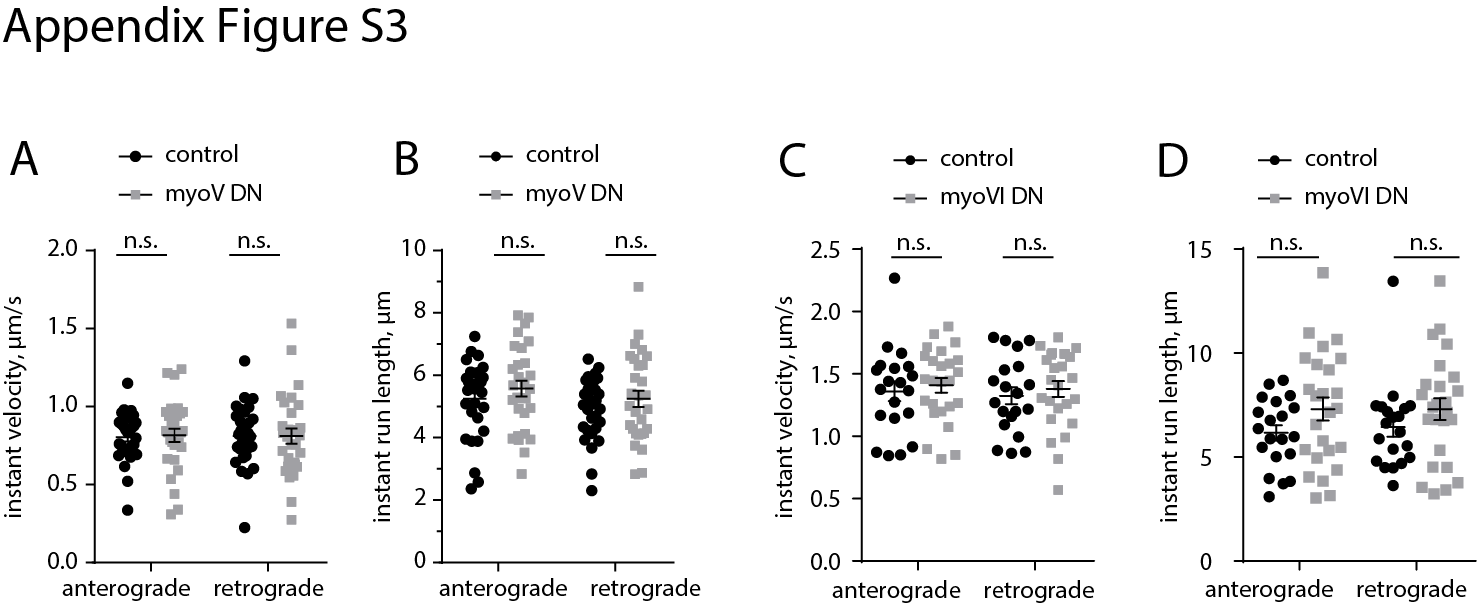


**Appendix Figure S3 – Effects of myosin V and myosin VI dominant-negative overexpression on LAMP1-mCherry motility.**

**A, B.** Further characterization of myoV DN effect on LAMP1-mCherry organelles (see also Fig. 7E-H**)**. MyoV DN does not change the instant velocity (**A**) or run length (**B**). 2-tailed unpaired Student’s t-test. Same dataset as Fig. 7E-H. Control: *n*=31 dendritic segments of 21 cells in 3 independent experiments. MyoV DN: *n* = 29 analysed dendritic segments from 21 cells in 3 independent cultures.

**C, D.** Further characterization of myoVI DN effect on LAMP1-mCherry organelles (see also Fig 7I-L**).** MyoVI DN does not change the instant velocity (**D**) or run length of lysosomes (**E**). 2-tailed unpaired Student’s t-test (**D**) and -tailed Mann-Whitney U-test (**E**). Same dataset as Fig 7I. *n*=20 (control), *n*=25 (myoVI DN) analysed dendritic segments of 20 cells (control) and 23 cells (myoVI DN) in 2 independent cultures. Data are presented as mean ± SEM.

**Appendix References**

Caussinus E, Kanca O, Affolter M (2011) Fluorescent fusion protein knockout mediated by anti-GFP nanobody. Nature structural & molecular biology 19**:** 117-121

Gee CE, Ohmert I, Wiegert JS, Oertner TG (2017) Preparation of Slice Cultures from Rodent Hippocampus. Cold Spring Harb Protoc 2017

Kapitein LC, Schlager MA, van der Zwan WA, Wulf PS, Keijzer N, Hoogenraad CC (2010) Probing intracellular motor protein activity using an inducible cargo trafficking assay. Biophysical Journal 99**:** 2143-2152

Kapitein LC, van Bergeijk P, Lipka J, Keijzer N, Wulf PS, Katrukha EA, Akhmanova A, Hoogenraad CC (2013) Myosin-V opposes microtubule-based cargo transport and drives directional motility on cortical actin. Curr Biol 23**:** 828-834

Katrukha EA, Mikhaylova M, van Brakel HX, van Bergen En Henegouwen PM, Akhmanova A, Hoogenraad CC, Kapitein LC (2017) Probing cytoskeletal modulation of passive and active intracellular dynamics using nanobody-functionalized quantum dots. Nature communications 8**:** 14772

Mikhaylova M, Bär J, van Bommel B, Schätzle P, YuanXiang P, Raman R, Hradsky J, Konietzny A, Loktionov EY, Reddy PP et al (2018) Caldendrin Directly Couples Postsynaptic Calcium Signals to Actin Remodeling in Dendritic Spines. Neuron 97**:** 1110-1125.e1114

Wiegert JS, Gee CE, Oertner TG (2017) Single-Cell Electroporation of Neurons. Cold Spring Harb Protoc 2017**:** pdb prot094904

González-Gallego J, Konietzny A, Perez-Alvarez A, Bär J, Maier U, Drakew A, Hammer JAI, Demmers JAA, Dekkers DHW, Frotscher M, Kneussel M, Oertner T, Wagner W & Mikhaylova M (2019) Characterization of neuronal synaptopodin reveals a myosin V-dependent mechanism of synaptopodin clustering at the post-synaptic sites. bioRxiv (10.1101/526509)
