# Supplementary material for: F‐actin patches associated with glutamatergic synapses control positioning of dendritic lysosomes
Source: EMBO J. 2019 Jun 27;38(15):e101183. doi: 10.15252/embj.2018101183 (PMC6669925; doi:10.15252/embj.2018101183)
Supplement: Supplementary file 2 — Expanded View Figures PDF [file EMBJ-38-e101183-s002.pdf]

Expanded View Figures

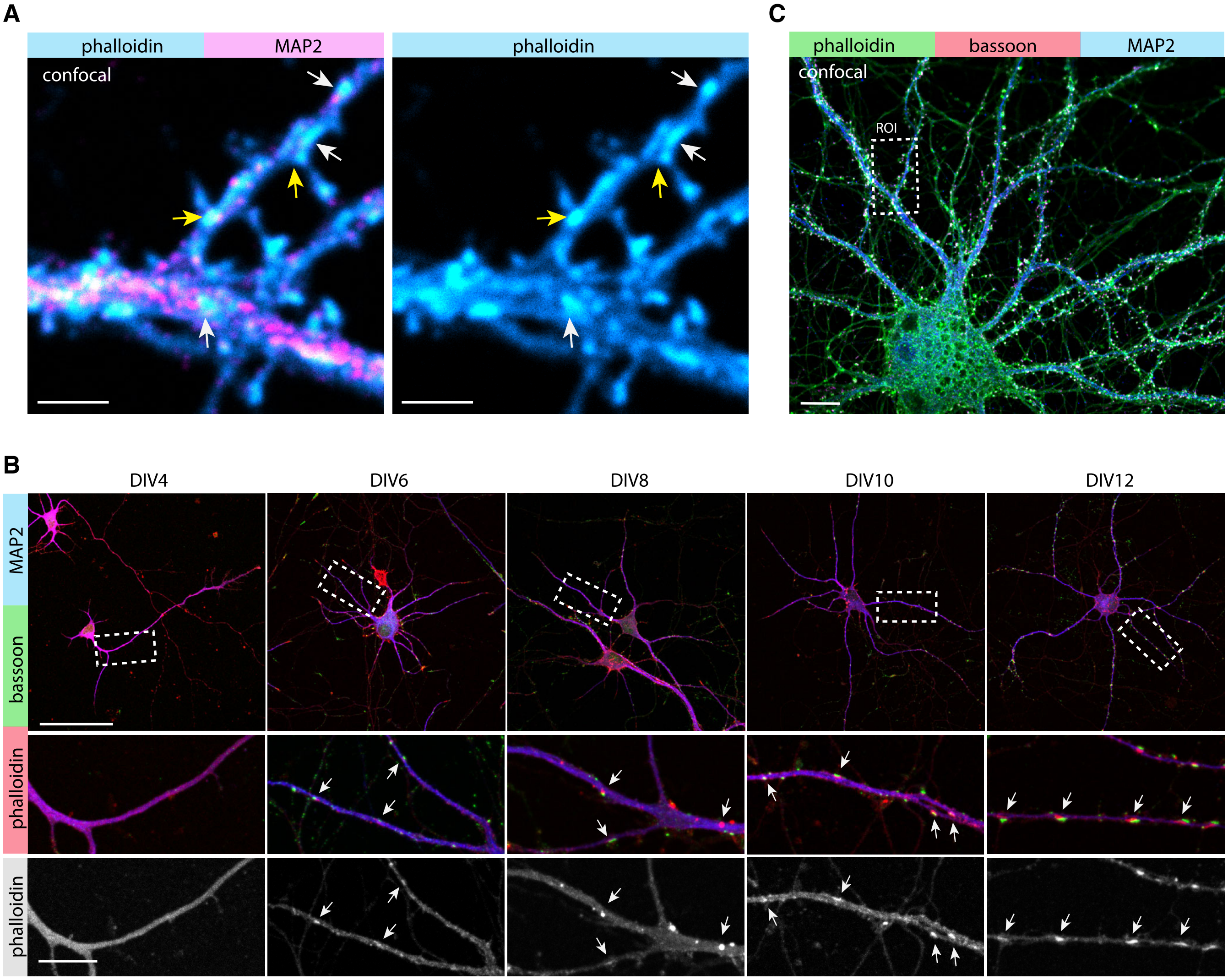

Figure EV1. Appearance of dendritic actin patches coincides with the development of synapses and maturation of primary cultures.

A Confocal image of the dendritic segment shown in Fig 1A. The segment is positive for the microtubule dendritic marker MAP2. The arrows indicate examples of actin patches at the base of dendritic spines (yellow arrows) and within the dendritic shaft (white arrows). Scale bar: 2 µm.

B Representative confocal images of the development of primary hippocampal cultures and zoom-in of a dendritic segment. Actin patches appear together with the development of presynaptic sites, positive for bassoon. Arrows indicate actin patches. Scale bar: 50 µm, 10 µm (Zoom-in).

C Confocal image of a hippocampal neuron stained for MAP2, bassoon, and F-actin labeled with phalloidin-Atto647N. The ROI indicates the dendritic segment shown in Fig 1C. Scale bar: 10 µm.

 © 2019 The Authors

Figure EV2. Excitatory shaft synapses in hippocampal primary and slice cultures.

A Upper panel: Maximum projection of a confocal image stack of a primary hippocampal neuron at DIV17, transfected with mRuby2 (cell fill) and FingR-PSD95-eGFP as an excitatory postsynaptic marker, and stained for bassoon as a presynaptic marker. Lower panel: Higher magnification image and corresponding 3D reconstruction show the presence of excitatory shaft synapses (arrows). Images are presented with a 1 px Gaussian blur filter. Scale bar: 10 μm, 5 μm.

B Upper panel: Maximum projection of a confocal image stack displaying the apical dendrite of a CA1 pyramidal neuron in a hippocampal slice culture at DIV18, electroporated with mRuby2 (cell fill) and FingR-PSD95-eGFP as excitatory postsynaptic marker. Lower panel: 3D reconstruction demonstrated the presence of excitatory shaft synapses (red). Images are presented with a 1 px Gaussian blur filter. See also Movie EV1. Scale bar: 10 μm, 5 μm (Zoom-in).

C Quantification of actin patch size in control (DMSO)-, LatA (5 μM for 30 min)-, CK666 (50 μM for 2 h)-, and SMIFH2 (30 μM for 90 min)-treated neurons. Patch size for spine-associated patches is not significantly changed after treatment. The size of shaft-associated patches was reduced after LatA treatment. One-way ANOVA with Dunnett's post hoc test. *$P = 0.02$. $n = 71$ (ctr), $n = 10$ (LatA), $n = 31$ (CK666), and $n = 11$ (SMIFH2) spine-associated patches and $n = 151$ (ctr), $n = 95$ (LatA), $n = 68$ (CK666), and $n = 28$ (SMIFH2) shaft-associated patches in 24, 17, 12, and 6 dendrites of 16, 15, 10, and six cells in two independent cultures. Data are presented as mean ± SEM.

D Quantification of normalized actin patch intensity as in (C). Normalized actin patch intensity for spine-associated patches was reduced in LatA (5 μM for 30 min)- and SMIFH2 (30 μM for 90 min)-treated groups. For shaft-associated patches, it was only reduced after LatA treatment. One-way ANOVA with Dunnett's post hoc test. *$P = 0.03$, ***$P < 0.001$. Same n as in (C). Data are presented as mean ± SEM.

E Representative STED and confocal images of DIV18 primary hippocampal neurons treated with brefeldin A (BFA, 100 ng/ml for 10 h), a drug that disrupts endosomes. Neurons were stained with α-MAP2 antibody and phalloidin-Atto647N, and arrows indicate examples of actin patches. BFA treatment did not affect the number of actin patches, nor alter the effect of CK-666 (Arp2/3 inhibitor) and SMIFH2 (formin inhibitor) on actin patches. Scale bar: 5 μm.

© 2019 The Authors

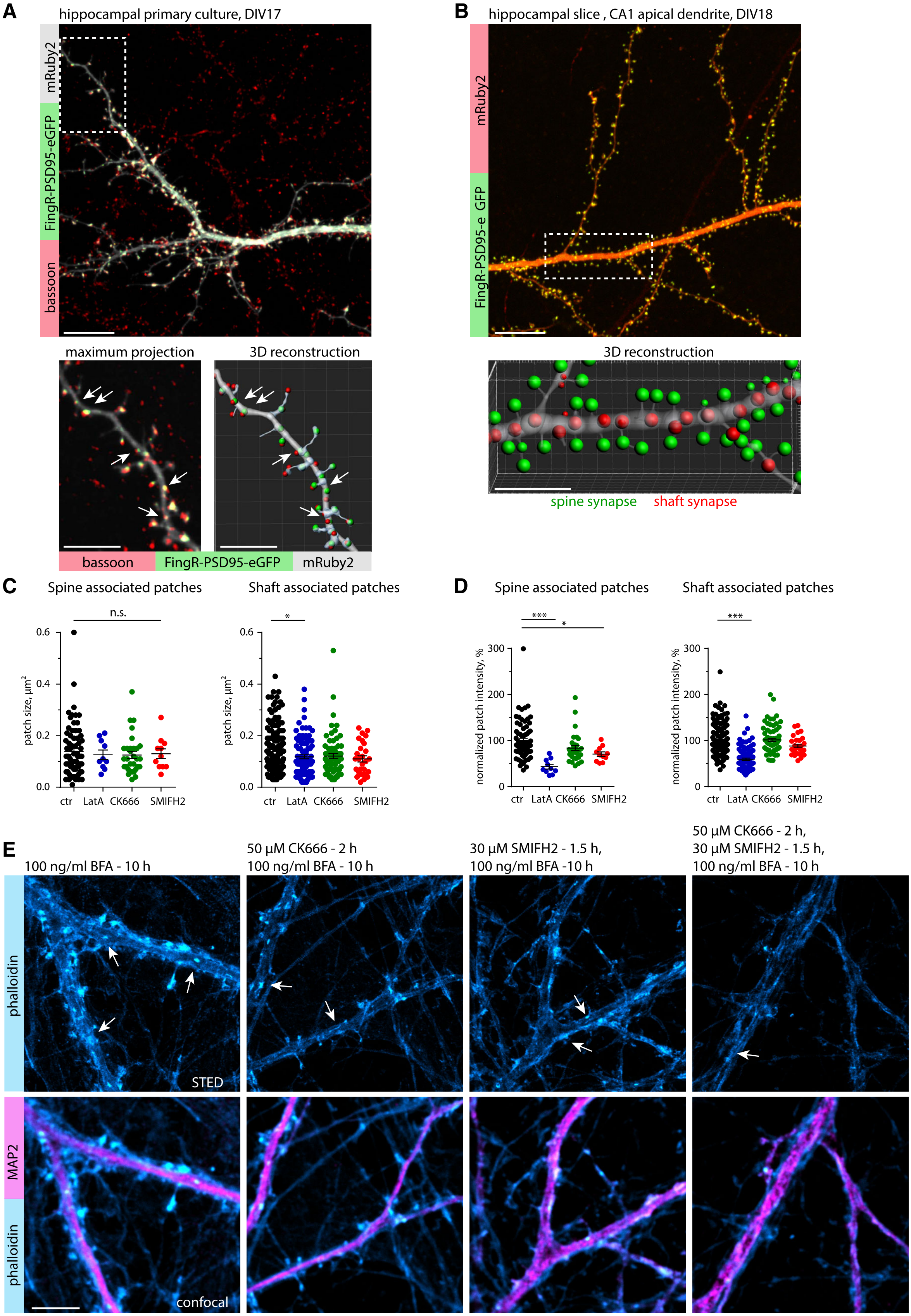

Figure EV2.

© 2019 The Authors

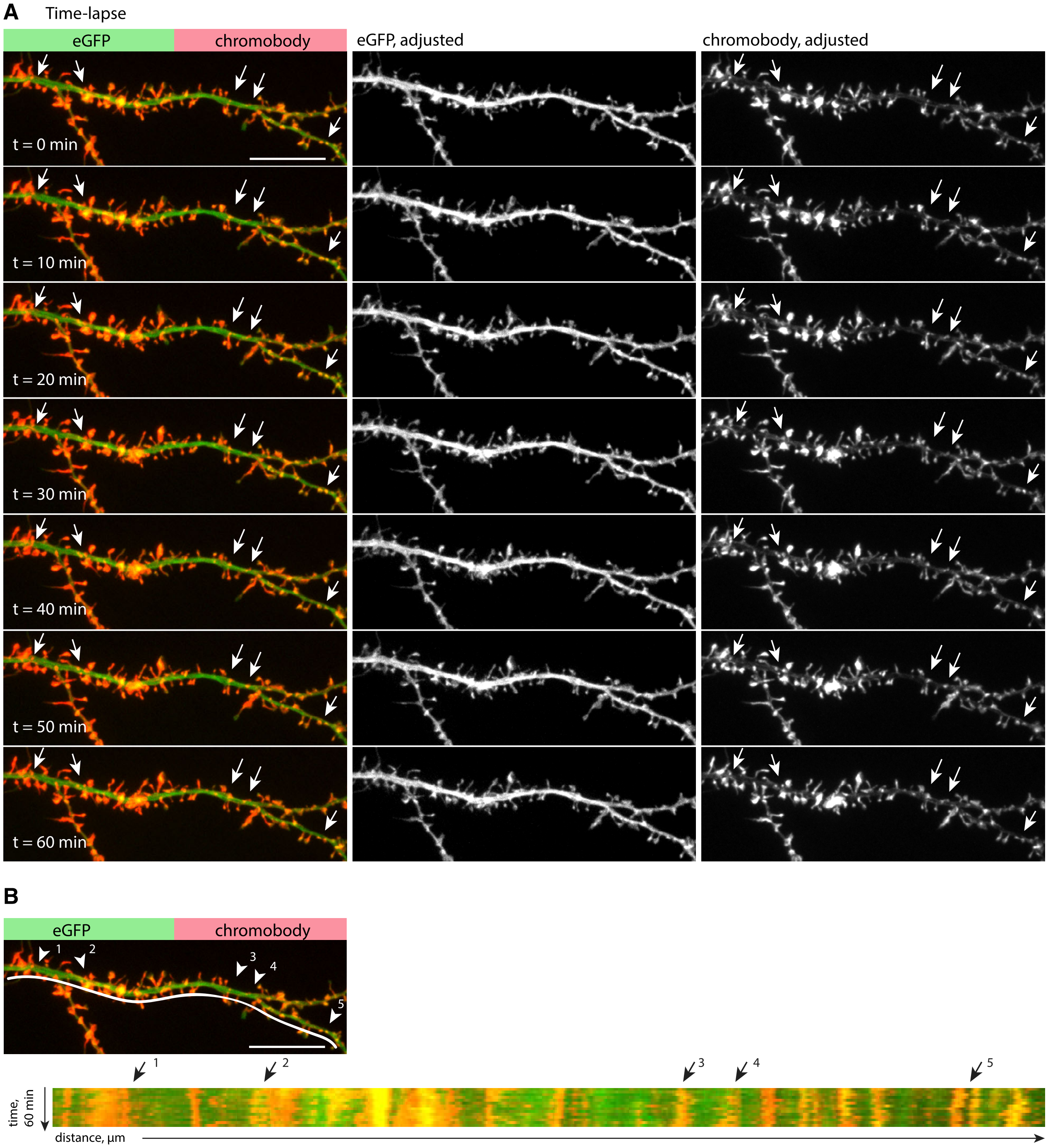

Figure EV3. Dendritic actin patches display high actin dynamics, but their existence and position are relatively stable.

A Individual stills from time-lapse spinning-disk confocal imaging of a dendritic segment of a DIV16 hippocampal neuron, expressing eGFP and chromobody-tagRFP. Actin patches (arrows) are stable over the entire imaging period (60 min). Images are compensated for bleaching and drift corrected. The lookup table of individual channels is adjusted for representation. Scale bar: 10 µm.

B Kymograph of the dendritic segment shown in (A). The presence and position of actin patches are relatively stable over a period of 60 min (arrows indicate examples of stable patches). Scale bar: 10 µm.

© 2019 The Authors

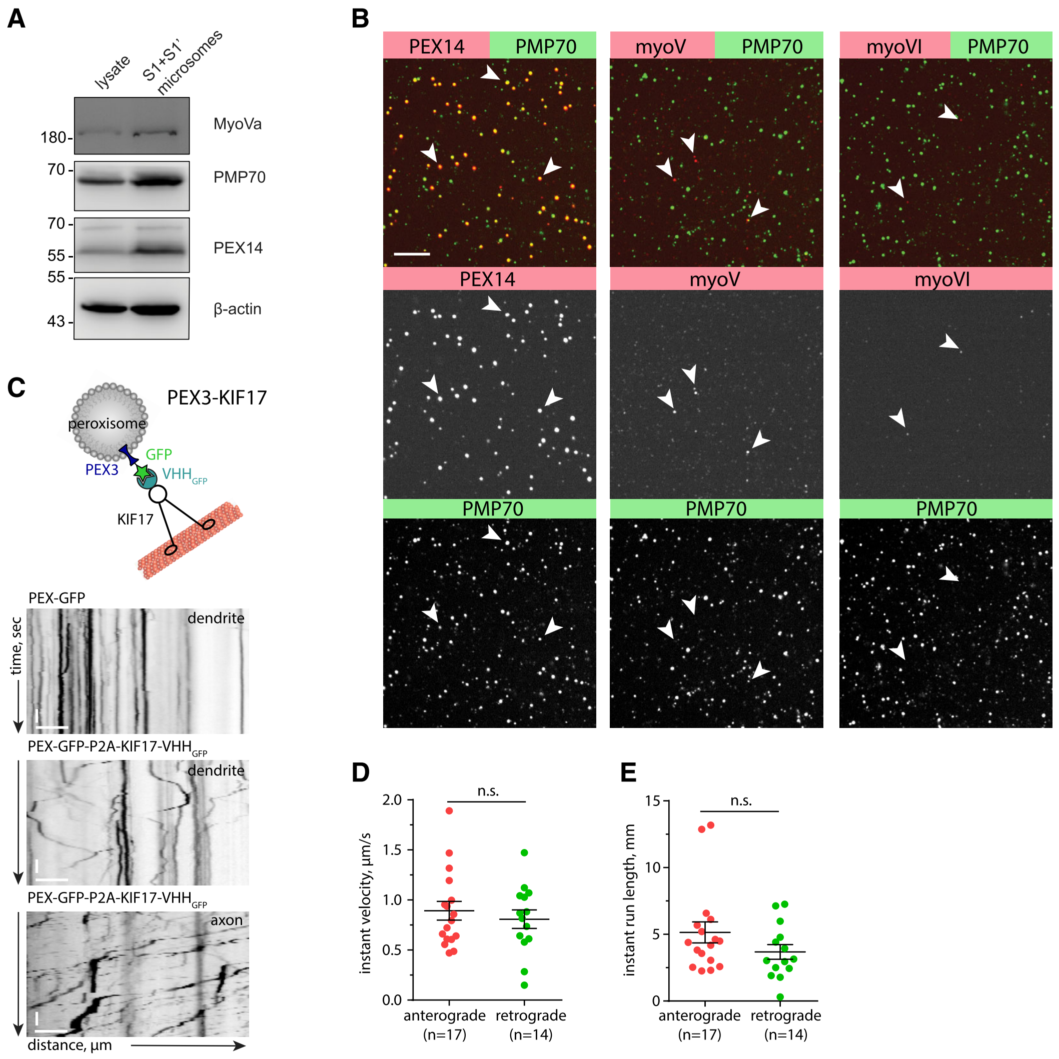

Figure EV4. Characterization of PEX-GFP-KIF17 construct for controlled cargo-motor assay in living neurons.

A Western blot of cleared cortical lysate (input) and enriched microsome fraction stained with antibodies against myosin Va (myoVa), PMP70, PEX14, and β-actin shows enrichment of peroxisomal markers PMP70 and PEX14, and the presence of myosin V (myoVa) in the fraction.

B Immunostaining of peroxisomes enriched from rat brain, identified with the peroxisome markers PEX14 and PMP70, is negative for myosin V (myoV) and myosin VI (myoVI). Scale bar: 5 µm. Data are presented as mean ± SEM.

C Upper panel: Schematic of a peroxisome coupled to constitutively active KIF17-VHH_{GFP} via PEX3-GFP. Both constructs are expressed on the same bicistronic P2A vector to ensure co-expression. Lower panel: representative kymographs from a time-lapse series of a DIV16 hippocampal neuron expressing PEX3-GFP (control; no motility) or PEX3-GFP-P2A-KIF17-$VHH_{GFP.}$ Bidirectional movement in dendrite and unidirectional movement (anterograde) in axon indicate the presence of KIF17 as the only processive microtubule-dependent motor. Scale bar: 2 µm, 10 s.

D, E Analysis of the instant velocity and instant run lengths of KIF17-coupled peroxisomes moving in the anterograde or retrograde direction (corresponding to Fig 6E–J). Peroxisomes transported by KIF17 move uniformly in both directions. Two-tailed unpaired Student's *t*-test. n = 14 (anterograde) and n = 17 (retrograde) of 17 dendritic segments of five cells in 1 culture. Data are presented as mean ± SEM.

 © 2019 The Authors

Figure EV5. Effects of myosin V and myosin VI pharmacological inhibitors on LAMP1-eGFP motility.

A Representative kymographs from dendritic segments of a DIV16 primary hippocampal neuron expressing LAMP1-eGFP, before (control) and after treatment with the myosin V inhibitor MyoVin (30 μM, 30 min). Scale bar: 5 μm, 15 s.

B Quantification of mobile and stationary lysosomes (LAMP1-eGFP). MyoVin treatment increased the number of stationary lysosomes, but did not significantly change the mobile fraction. Two-tailed paired Student's *t*-test. ***$P < 0.001$. n = 25 dendritic segments of 21 neurons in three independent cultures.

C Quantification of lysosome motility from kymographs as shown in (A). Analyzed was the total time spent pausing (< 1 min), stationary (≥ 1 min), or moving in the anterograde or retrograde direction. MyoVin increased stationary time and reduced short-term pausing. RM-2-ANOVA with lysosome behavior and treatment as within-group factors. $F(3, 72) = 14.623$, $P < 0.001$ with Newman–Keuls post hoc test **$P = 0.001$, ***$P < 0.001$. Same n as in (B). Data are presented as mean ± SEM.

D MyoVin treatment increased the cumulative peroxisome pausing time. Wilcoxon signed rank test. **$P = 0.005$. Same n as in (B). Data are presented as mean ± SEM.

E Cumulative frequency of the duration of pausing events of lysosomes (LAMP1-eGFP) in control (DMSO) and the presence of MyoVin. Fully stationary lysosomes were excluded. The presence of MyoVin did not change the pausing time distribution. Two-tailed Mann–Whitney *U*-test. n = 782 (control) and n = 654 (MyoVin) events in 25 dendritic segments of 21 cells in three independent culture.

F Analysis of the instant velocity (left) and instant run lengths (right) of lysosomes (LAMP1-eGFP) moving in the anterograde or retrograde direction. MyoVin treatment decreased instant velocities. Paired two-tailed Student's *t*-test (mobile) or Wilcoxon matched paired (stationary) *$P = 0.012$, **$P = 0.0029$. Same n as in (B).

G Representative kymographs from a dendritic segment of a DIV16 primary hippocampal neuron expressing LAMP1-eGFP, before (control) and after treatment with the myosin VI inhibitor TIP (4 μM, 30 min). Scale bar: 5 μm, 15 s.

H Quantification of mobile and stationary lysosomes (LAMP1-eGFP). TIP has no significant effect on lysosome numbers. Paired two-tailed Student's *t*-test (stationary) or Wilcoxon matched pairs test. n = 22 dendritic segments of 19 cells in three independent cultures.

I Quantification of lysosome motility from kymographs as shown in (G). Analyzed was the total time spent pausing (< 1 min), stationary (≥ 1 min), or moving in the anterograde or retrograde direction. Treatment with TIP slightly increased the long-term (≥ 1 min) stationary fraction. RM-2-ANOVA with lysosome behavior and treatment as within-group factors. $F(3, 63) = 3.46$, $P = 0.021$ with Newman–Keuls post hoc test. *$P = 0.01$. Same n as in (H). Data are presented as mean ± SEM.

J TIP treatment did not significantly change the summed pausing time of lysosomes (LAMP1-eGFP). One-sample *t*-test against 100%. Same n as in (H). Data are presented as mean ± SEM.

K Cumulative frequency of the duration of pausing events of lysosomes (LAMP1-eGFP) in control (DMSO) and in the presence of TIP. Fully stationary lysosomes were excluded. TIP treatment did not affect the distribution of pausing events. Two-tailed Mann–Whitney *U*-test. n = 1029 (control) and n = 908 (TIP) events of 22 dendritic segments of 19 cells in three independent cultures.

L Analysis of the instant velocity (left) and the instant run lengths (right) of lysosomes (LAMP1-eGFP) moving in the anterograde or retrograde direction. TIP treatment decreased the instant velocity and run length in both directions. Two-tailed Wilcoxon matched pairs test (anterograde velocity) ***$P = 0.0003$ or paired two-tailed Student's *t*-test, *$P = 0.03$ (anterograde run length), *$P = 0.04$ (retrograde run length), ***$P < 0.001$ (retrograde velocity). Same n as in (H).

© 2019 The Authors

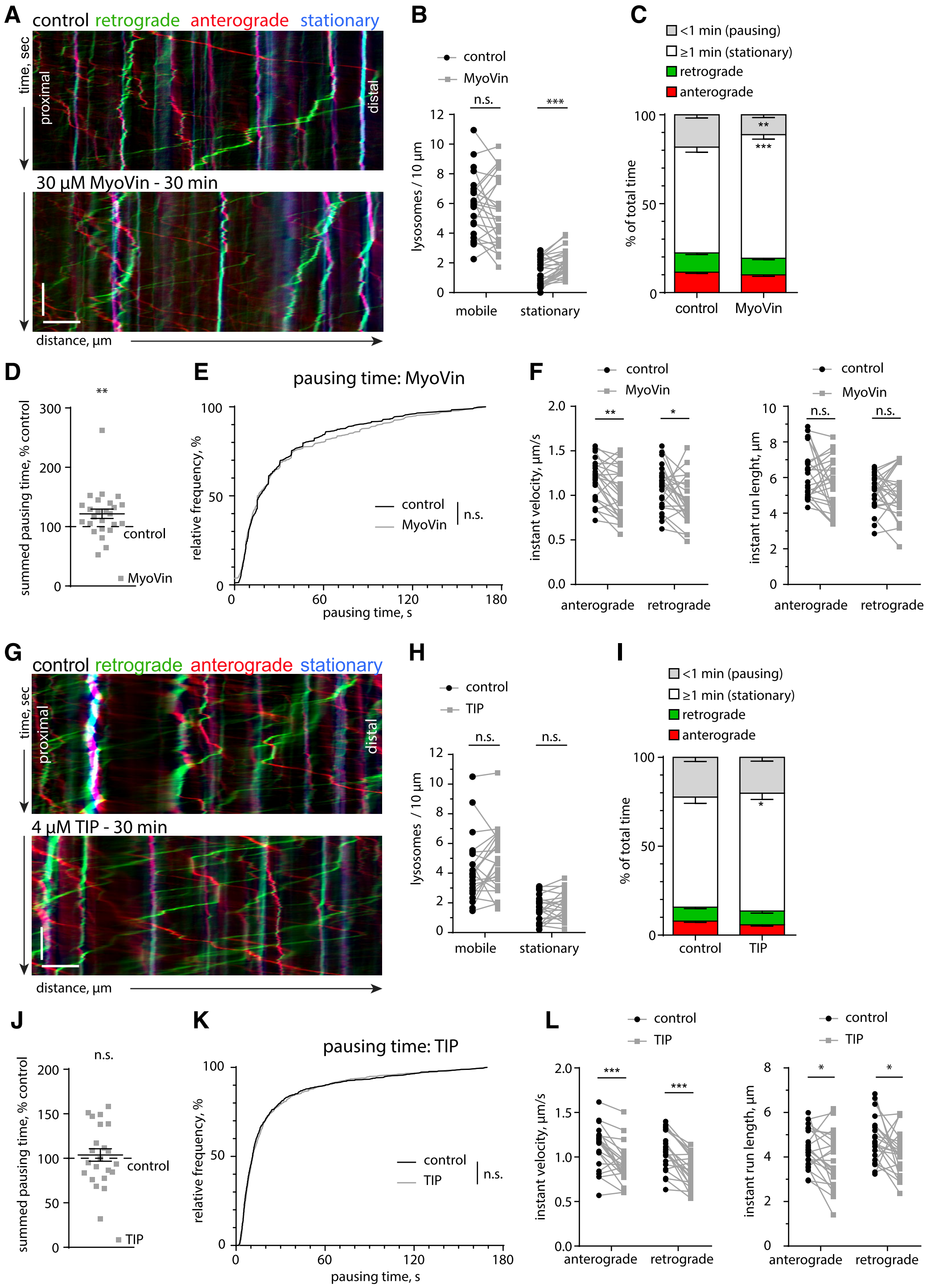

Figure EV5.

© 2019 The Authors
