# Supplementary material for: F‐actin patches associated with glutamatergic synapses control positioning of dendritic lysosomes
Source: EMBO J. 2019 Jun 27;38(15):e101183. doi: 10.15252/embj.2018101183 (PMC6669925; doi:10.15252/embj.2018101183)
Supplement: Supplementary file 3 — Movie EV1 [file EMBJ-38-e101183-s003.zip › Movie_EV1/Movie_EV1.docx]

**Movie EV1. Excitatory shaft synapses in hippocampal slice culture.** Refers to Figure EV2. Rotation of CA1 apical dendrite and reconstruction shown in Fig EV2B lower panel.
