# Supplementary material for: F‐actin patches associated with glutamatergic synapses control positioning of dendritic lysosomes
Source: EMBO J. 2019 Jun 27;38(15):e101183. doi: 10.15252/embj.2018101183 (PMC6669925; doi:10.15252/embj.2018101183)
Supplement: Supplementary file 5 — Movie EV3 [file EMBJ-38-e101183-s005.zip › Movie_EV3/Movie_EV3.docx]

**Movie EV3. Time-lapse imaging of hippocampal neuron transfected with actin-chromobody and LAMP1-GFP.** Refers to Figure 6. Imaged at 4 frames per second, played at 40 frames per second.
