# Supplementary material for: F‐actin patches associated with glutamatergic synapses control positioning of dendritic lysosomes
Source: EMBO J. 2019 Jun 27;38(15):e101183. doi: 10.15252/embj.2018101183 (PMC6669925; doi:10.15252/embj.2018101183)
Supplement: Supplementary file 6 — Movie EV4 [file EMBJ-38-e101183-s006.zip › Movie_EV4/Movie_EV4.docx]

**Movie EV4. Time-lapse imaging of hippocampal neuron transfected with actin-chromobody and PEX-KIF17.** Refers to Figure 6. Imaged at 3 frames per second, played at 30 frames per second.
